# Utilization of Away-From-Home Food Establishments, Dietary Approaches to Stop Hypertension Dietary Pattern, and Obesity

**DOI:** 10.1016/j.amepre.2017.06.003

**Published:** 2017-11

**Authors:** Tarra L. Penney, Nicholas R.V. Jones, Jean Adams, Eva R. Maguire, Thomas Burgoine, Pablo Monsivais

**Affiliations:** 1 UK Clinical Research Collaboration Centre for Diet and Activity Research, Medical Research Council Epidemiology Unit, University of Cambridge School of Clinical Medicine, Cambridge, United Kingdom; 2 Department of Nutrition and Exercise Physiology, Elson S. Floyd College of Medicine, Washington State University, Spokane, Washington

## Abstract

**Introduction:**

Eating meals away from home has been associated with the consumption of unhealthy foods and increased body weight. However, more rigorous assessment of the contribution of different types of away-from-home food establishments to overall diet quality and obesity is minimal. This study examined usage of these food establishments, accordance to the Dietary Approaches to Stop Hypertension (DASH) dietary pattern and obesity status in a nationally representative sample of adults in the United Kingdom.

**Methods:**

A cross-sectional analysis of data from a national survey (N=2,083 aged ≥19 years, from 2008 to 2012) with dietary intake measured using a 4-day food diary, and height and weight measured objectively. Exposures included usage of (i.e., by proportion of energy) all away-from-home food establishments combined, and fast-food outlets, restaurants, and cafés separately. Outcomes included accordance with the DASH diet, and obesity status. Multivariable logistic regressions were conducted in 2016 to estimate associations between food establishments, diet quality, and obesity.

**Results:**

People consuming a higher proportion of energy from any away-from-home food establishment had lower odds of DASH accordance (OR=0.45, 95% CI=0.31, 0.67) and increased odds of obesity (OR=1.48, 95% CI=1.10, 1.99). After adjustment, only use of fast-food outlets was significantly associated with lower odds of DASH accordance (OR=0.48, 95% CI=0.33, 0.69) and higher odds of obesity (OR=1.30, 95% CI=1.01, 1.69).

**Conclusions:**

Although a greater reliance on eating away-from-home is associated with less-healthy diets and obesity, dietary public health interventions that target these food establishments may be most effective if they focus on modifying the use of fast-food outlets.

## Introduction

Poor diet and obesity are global epidemics that present a significant challenge for public health and the prevention of chronic disease.^1^ In the United Kingdom (UK), diet is the leading behavioral contributor to disease burden,^2^ with poor diet quality associated with an increased risk of cardiovascular disease,^3^ diabetes,^4^ certain types of cancer,^5^ and other chronic conditions, such as overweight and obesity.^6^ Obesity (a BMI ≥30kg/m^2^) in particular is considered a global epidemic with a doubling of prevalence worldwide since 1980, reaching approximately 600 million adults as of 2014.^7^ In the UK, approximately 26% of men (up from 13.2% in 1993) and 23.8% of women (up from 16.4%) are living with obesity.^8^

Dietary risk factors for obesity and chronic disease include a low intake of fruits and vegetables, whole grains, low-fat milk, fiber, nuts, and seeds, and high intake of red and processed meat, sugar, and sodium^9^; these poor dietary patterns are common in the UK population.^2^ In comparison, diets rich in fruits and vegetables and low-fat dairy products, and relatively low in fats and sugars, are consistent with dietary patterns that are protective against disease risk.^9^ Many characterizations of diet quality are relevant for understanding dietary risk for chronic disease, such as the Healthy Eating Index, Alternative Healthy Eating Index, and the Mediterranean Diet Score.^10^ In addition, the Dietary Approaches to Stop Hypertension (DASH) dietary pattern provides strong experimental evidence that adherence can improve health outcomes. Randomized trials showed that adherence to a DASH diet can markedly reduce blood pressure,^11^, ^12^ whereas observational studies showed that DASH-accordant diets were associated with reduced weight gain,^13^ lower incidence of stroke,^14^ and a reduced risk of type 2 diabetes.^15^ Therefore, the DASH dietary pattern is the focus of this study.

Improving overall diet quality within the population requires understanding a complex set of determinants.^16^ One factor that may adversely affect diet quality and promote obesity at the population level is a reliance on eating away from home. Over the past few decades, frequency of meals consumed away from home has been increasing,^17^ accounting for a growing proportion of daily energy intake across all age groups.^18^, ^19^, ^20^ Systematic reviews have suggested that greater frequency of eating away from home is associated with poorer nutrient and dietary intake^21^ and increased adiposity and weight gain,^22^ possibly due to the large portions of energy-dense foods available in different food establishments.^23^ However, away-from-home food establishments represent a diverse set of food services, some focusing on slow or fast service and catering to different consumer preferences.^24^ Much of the evidence to date treats this collection of away-from-home food establishments as homogeneous in their association with poor diet or health,^25^, ^26^, ^27^, ^28^ often employing retrospective measurement unable to explore differential associations between use and diet quality.^29^, ^30^, ^31^ Moreover, many studies of dietary outcomes focus on individual nutrients (e.g., fat, cholesterol, sodium),^21^, ^32^ or foods (e.g., meat, takeaway, fruits, and vegetables)^33^ rather than measures of overall diet quality.

The aim of this work is to employ a prospective assessment of energy intake from all away-from-home food establishments, and restaurants, fast-food outlets, and cafés specifically, to examine independent association(s) with overall diet quality and obesity.

## Methods

### Study Sample

This work was a cross-sectional analysis of nationally representative dietary surveillance data for adults aged ≥19 years from the UK.

Adults (N=2,083) aged ≥19 years from Year 1 to 4 (2008 to 2012) of the UK National Diet and Nutrition Survey (NDNS) rolling program were used (February 2015 release).^34^ NDNS is a yearly cross-sectional survey collecting information on the food consumption, nutrient intake, and nutritional and health status of individuals living in the UK. Sampling, recruitment, and data collection methods are constant from year to year to allow data to be combined across survey years. A detailed description of the multi-stage stratified random sampling procedure and design has been reported elsewhere.^9^ In short, sampling for each wave was based on the random selection of postcode sectors across the UK, followed by the selection of households, and lastly the selection of households with up to one adult and one child (Appendix, available online). Data collection involved a researcher interview collecting sociodemographic variables, the completion of a 4-day food diary, and a nurse visit including measurement of height and weight.

Overall, 91% of households eligible for inclusion agreed to take part in Waves 1–4 of NDNS. Usable food diaries (3 or 4 days completed) were collected from at least one household member in 58% of eligible households. At the individual level, 56% of those selected to take part completed usable food diaries, including 2,083 adults.^9^ NDNS was approved by Oxfordshire Research Ethics Committee and written informed consent was obtained from all participants.^9^

### Measures

At the initial interviewer visit, participants were given instructions to record all food and beverages consumed in and out of the home over 3 or 4 consecutive days using unweighed food diaries. Portion sizes were estimated using household measure and weights from food package labels obtained online, by purchase, or from participants. At the final interviewer visit, the food diaries were checked for missing information and further detail was added before being returned to the Medical Research Council–Human Nutrition Research site for coding. The diaries were coded by trained assistants using the in-house dietary assessment software, Diet In Nutrients Out, with nutrient values provided by the UK NDNS Nutrient Databank.^35^

Regarding food establishment identification, the NDNS applied a form of ecological momentary assessment to collect reports on eating events in real-time throughout the daily lives of participants to reduce recall bias.^36^ Within the 4-day food diary, each recording day was divided into seven timeslots (6:00am–9:00am, 9:00am–12:00pm, 12:00pm–2:00pm, 2:00pm–5:00pm, 5:00pm–8:00pm, 8:00pm–10:00pm, and 10:00pm–6:00am). In each timeslot, participants could report multiple food items and the location where each food was consumed. Food establishment classification was done by NDNS using Diet In Nutrients Out and a set of classification criteria (Appendix, available online). The authors used the pre-existing categories for three away-from-home food establishments—“restaurants, pubs, and night clubs,” “fast-food and takeaway,” and “cafés and sandwich shops”—and collapsed all remaining categories into “other” away-from-home locations and “home.” Eating occasions were defined as a group of food items consumed by a participant within a single sitting^37^ on the same diary day, within the same timeslot, and at the same location classification (Appendix, available online).

The proportion of total energy intake (kJ) from all locations was calculated. Exposures included three away-from-home food establishments—“sit-down restaurants,” “fast-rood outlets,” and “cafés”—and “other” locations away from the home with corresponding total energy intake for each participant. Proportion of energy intake was then calculated for all categories (energy intake within category/total energy intake), and then converted into levels of exposure. Tertiles (low, middle, high) were used for the total combined “away-from-home” (i.e., food establishment and other locations) exposure. Food establishment subcategories were dichotomized to provide an indicator of participant use (none, any) for “restaurants, pubs, and night clubs,” “fast-food and takeaway,” and “cafés and sandwich shops.”

Overall diet quality was assessed by quantifying accordance to the DASH dietary pattern using an established index.^14^, ^38^ The score is based on consumption of eight food groups and nutrients including fruits, vegetables, nuts and legumes, whole grains, low-fat dairy, red and processed meats, non-milk extrinsic sugars, and sodium, adjusted for energy using the residual method.^39^ The eight DASH food groups and associated scoring are presented and described in the Appendix (available online) with additional details on assessing accordance to DASH (i.e., the highest quintile). This characterization has been used previously in epidemiologic studies of the DASH diet in relation to cardiovascular disease and colorectal cancer.^38^, ^40^

Trained interviewers collected measurements of height and weight during participant nurse visits. Participants were measured in minimal clothing and without shoes. BMI was calculated from measured height and weight and categorized as obese (BMI ≥30 kg/m^2^) or not.^41^

Self-report survey questions were used to assess demographic factors including age (continuous) and sex. SES was represented using one indicator that was found to be patterned by both exposure and outcome variables. Total household income was equivalized for household composition and categorized as “£14,999 or below,” “£15,000–£24,999,” “£25,000–£34,999,” “£35,000–£49,999,” and “£50,000 and above.” Smoking status was categorized as “current smoker,” “ex-regular smoker” or “never regular smoker.” Survey year was categorized based on study year (1 to 4). Missing covariate values were also examined across all exposure variables, with no significant differences in percentages across exposure levels, then categorized for each variable and included in appropriate models to avoid case deletion (i.e., missing indicator approach).^42^

### Statistical Analysis

Descriptive statistics were used to summarize demographic, socioeconomic, behavioral, diet, health, and eating occasion variables across all away-from-home locations and specific food establishment exposures. Study weights, prepared by NDNS and provided with the data, were used to account for participant nonresponse; therefore weighted mean percentages (with 95% CIs) are presented rather than raw frequencies.

Binary logistic regressions were used to evaluate DASH accordance and obesity status by tertile of proportion of energy intake consumed away from home (Model 1). These models were then adjusted for demographic variables (age and sex) and other covariates (total energy [kJ], survey year, and smoking status [for obesity outcome only]) (Model 2), followed by socioeconomic status (income) (Model 3). In secondary analyses, logistic regression models were fitted to base models (Model 1, Model 2, and Model 3) replacing “away-from-home” exposure with “restaurants, pubs, and night clubs,” “fast-food and takeaway,” and “cafés and sandwich shops” respectively, mutually adjusting for each respective away-from-home food establishment and “other.” The resulting ORs from the secondary analyses for DASH accordance and obesity were interpreted as independent associations of the specific food establishment exposure being examined. NDNS analytic weights were used to ensure that analyses accounted for nonresponse bias and the survey’s complex sampling structure. All statistical analyses were carried out in Stata, version 14, in 2016. Sensitivity analyses examined exposures characterized using eating occasions in place of energy intake, alternative socioeconomic indicators (education and income combined), and examining the influence of total energy as a covariate on DASH.

## Results

The overall unweighted sample included N=2,083 adults (*n*=901, 43.2% men) and has been described in detail elsewhere.^9^ In short, the weighted sample is considered a demographically representative sample of the UK population covering a range of socioeconomic strata (Appendix, available online, provides descriptive statistics of the full sample).

Table 1 and Table 2 present selected sample demographic, socioeconomic, behavioral, diet, and weight-related characteristics across different away-from-home food establishment exposures. Table 1 shows that greater away-from-home usage (i.e., all food establishments and other locations) includes sample members who are younger, belonged to the most socioeconomically advantaged groups (higher education, income, and occupational status), smoked less, had less healthy dietary intakes (lower fruit and vegetable consumption, lower DASH accordance, and more total energy intake [kJ/day]), and a normal weight. Table 2 shows that sample members in the most advantaged socioeconomic groups were more likely to be sit-down restaurant and café users; however, that pattern was reversed for fast-food users who were less socioeconomically advantaged. For all food establishment categories, a higher percentage of users had a normal BMI.

**Table 1:** Weighted Sample Characteristics by Proportion of Energy (kJ) from All Away-From-Home Food Establishment Use

	**Tertile of away-from-home usage**			
**Characteristic**	**Lowest**	**Middle**	**Highest**	**Total**
*n*	708	706	669	2,083
Proportion of energy (kJ) (min–max)	0.00 – 0.14	0.14 – 0.34	0.34 – 1.0	0 – 1
Demographic				
Age, years	56.2	47.9	40.0	48.0
Sex (% male)	47.5	44.6	53.5	48.5
Ethnicity (% white)	88.9	91.3	88.4	89.6
Socioeconomic				
Educational attainment (% degree)	16.1	22.8	31.7	23.5
Equalized income (% >£35,000)	22.5	30.6	41.6	31.8
Occupation (% professional)	34.6	44.7	48.0	42.4
Behavior				
Smoking (% never smoked)	53.4	55.8	57.1	55.4
Diet				
Fruit and vegetable (g/day)^a^	313 (298, 328)	288 (273, 302)	266 (253, 280)	289 (280, 298)
DASH score (% accordant)	21.2	16.8	11.7	16.6
Energy intake (kJ/day)^a^	7,262 (7,074, 7,455)	7,568 (7,333, 7,802)	8,284 (8,058, 8,505)	7,706 (7,576, 7,832)
Adiposity				
% Normal BMI (18–25 kg/m^2^)	28.8	30.4	33.4	30.9

a Weighted mean % (95% CI). DASH accordant = highest quintile. DASH, Dietary Approaches to Stop Hypertension.

**Table 2:** Weighted Sample Characteristics by Proportion of Energy (kJ) for Sit-down Restaurant, Fast-food, or Café Use

	**Food establishment use**					
	**Sit-down restaurant**		**Fast food**		**Café**	
**Characteristic**	**None**	**Any**	**None**	**Any**	**None**	**Any**
*n*	1,399	684	1,569	514	1,675	408
Proportion of energy (kJ) (≤max)	0.00	≤0.85	0.00	≤0.73	0.00	≤0.30
Demographic						
Age, years	48.7	46.7	50.6	40.8	47.8	49.1
Sex (% male)	47.3	50.9	47.7	50.7	49.8	42.7
Ethnicity (% white)	88.9	90.9	89.7	89.2	88.5	94.3
Socioeconomic						
Educational attainment (% degree)	20.5	29.4	23.9	22.6	22.7	27.4
Equalized income (% >£35,000)	28.3	38.5	33.2	28.0	30.3	38.6
Occupation (% professional)	40.4	46.3	43.1	40.7	41.4	47.4
Behavior						
Smoking (% never smoked)	53.4	59.4	56.1	53.6	54.6	59.3
Diet						
Fruit and vegetable (g/day)^a^	289 (278, 300)	289 (275, 303)	305 (295, 315)	244 (229, 260)	287 (277, 297)	299 (282, 316)
DASH score (% most accordant)	17.2	15.3	19.3	8.9	15.9	19.6
Energy intake (kJ/day)^a^	7, 541 (7,292, 7,610)	8,204 (7,999, 8,405)	7,467 (7,342, 7,597)	8,363 (8,037, 8,690)	7,668 (7,526, 7,806)	7,873 (7,626, 8,120)
Adiposity						
% Normal BMI (18–25 kg/m^2^)	30.0	32.6	30.0	33.2	30.4	32.8

a Weighted mean % (95% CI). DASH accordant = highest quintile. DASH, Dietary Approaches to Stop Hypertension.

**Figure 1:**
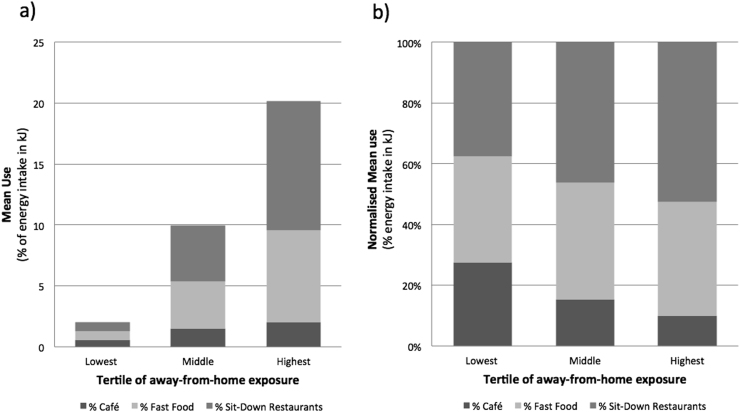
(a) Stacked weighted mean percentage of food establishment use by tertile of total away-from-home exposure. (b) Normalized weighted mean percentage contribution of combined food establishment use, also by tertile of total away-from-home exposure.

Across tertiles of away-from-home exposure, absolute usage of food establishments ranged from less than 5% to more than 20% of dietary energy intake (Figure 1a). The relative contribution of the three food establishment categories also varied across tertiles, with sit-down restaurants the dominant source of energy intake in the highest tertile (Figure 1b).

Regression analyses (Table 3) showed that after adjustment for known confounders, level of away-from-home usage was associated with lower odds of DASH accordance (OR=0.70, 95% CI=0.52, 0.95 and OR=0.45, 95% CI=0.31, 0.67, respectively) and higher odds of obesity (OR=1.41, 95% CI=1.06, 1.89 and OR=1.48, 95% CI=1.10, 1.99, respectively). Subsequent analyses revealed that away-from-home food establishment use and outcomes were differentially patterned by type of food establishment. After adjustment, fast-food use was associated with lower odds of DASH accordance (OR=0.48, 95% CI=0.33, 0.69) and higher odds of obesity (OR=1.30, 95% CI=1.01, 1.69). In Model 1, sit-down restaurant use was associated with lower odds of obesity (OR=0.73, 95% CI=0.58, 0.93), but these associations were attenuated when adjusted in Model 2, and then nonsignificant after adjustment for socioeconomic factors in Model 3 (0.81 [0.64–1.03]). Associations between café use, DASH accordance, and obesity remained null across all models. Sensitivity analyses provided no significant difference in the result reported.

**Table 3:** ORs and 95% CIs for DASH Accordance and Obesity Outcomes

	**Odds of DASH accordance**^a^**(*****n*****=2,083)**			**Odds of obesity**^b^**(*****n*****=1,902)**		
**Exposure**	**Model 1**^c^	**Model 2**^d^	**Model3**^e^	**Model 1**^c^	**Model 2**^d^	**Model 3**^e^
Tertile of away-from-home usage						
Lowest	1.00 (–)	1.00 (–)	1.00 (–)	1.00 (–)	1.00 (–)	1.00 (–)
Middle	0.75 (0.56, 1.01)	0.80 (0.60, 1.08)	0.70* (0.52, 0.95)	1.18 (0.9, 1.54)	1.33* (1.01, 1.76)	1.41* (1.06, 1.89)
Highest	0.49** (0.34, 0.69)	0.59** (0.41, 0.84)	0.45** (0.31, 0.67)	1.10 (0.77, 1.32)	1.34* (1.00, 1.79)	1.48** (1.10, 1.99)
Food establishment use^f^						
Sit-down restaurant						
None	1.00 (–)	1.00 (–)	1.00 (–)	1.00 (–)	1.00 (–)	1.00 (–)
Any	0.87 (0.64, 1.18)	0.89 (0.65, 1.2)	0.78 (0.56, 1.07)	0.73*(0.58, 0.93)	0.78*(0.61, 0.99)	0.81 (0.64, 1.03)
Fast food						
None	1.00 (–)	1.00 (–)	1.00 (–)	1.00 (–)	1.00 (–)	1.00 (–)
Any	0.41** (0.29, 0.58)	0.44** (0.30, 0.64)	0.48** (0.33, 0.69)	1.08 (0.84, 1.40)	1.34* (1.02, 1.74)	1.30* (1.01, 1.69)
Café						
None	1.00 (–)	1.00 (–)	1.00 (–)	1.00 (–)	1.00 (–)	1.00 (–)
Any	1.29 (0.94, 1.77)	1.27 (0.92, 1.75)	1.14 (0.81, 1.61)	0.86 (0.65, 1.15)	0.85 (0.63, 1.14)	0.88 (0.65, 1.18)

*Note:* Boldface indicates statistical significance (*p<0.05; **p<0.01).

a Dietary Approaches to Stop Hypertension (DASH) accordance is being in the highest quintile of adherence to the DASH dietary pattern.

b Obesity included participants with a BMI ≥30 kg/m^2^.

c Model 1: Unadjusted model.

d Model 2: Adjusted for age, sex, total energy (kJ), survey year (and smoking status for obesity models).

e Model 3: Additionally adjusted for SES.

f Model 3 additionally adjusted for proportion of energy from restaurant, fast food, café, and other away from home non-retail locations (as appropriate).

## Discussion

Determining if the use of specific away-from-home food establishments contributes to overall diet quality is important for informing interventions to improve diet and reduce obesity at the population level. Using prospective assessment of away-from-home eating, these analyses confirmed some findings of previous studies.^25^, ^26^, ^43^ Specifically, greater away-from-home eating was inversely associated with diet quality and positively associated with obesity, with those in the highest exposure group showing 55% lower odds of a DASH-accordant diet and 48% higher odds of obesity.

Going beyond previous research, usage of three different away-from-home food establishments including sit-down restaurants, fast-food outlets, and cafés were characterized using 4-day food diaries. This study revealed differential associations with overall diet quality and obesity depending on food establishment and adjustment for confounding factors. Fast-food outlet usage was the only food establishment significantly and inversely associated with diet quality (52% lower odds) and positively associated with obesity (30% higher odds), regardless of model adjustment. Therefore, although eating outside the home may be associated with a poor diet and excess weight,^43^ the authors’ examination of food establishments revealed that these associations could be mostly attributed to fast-food use independent of other food establishment usage, specifically sit-down restaurant and café use. Additionally, whereas other studies have examined away-from-home eating in relation to intake of specific foods or nutrients, this study examined usage of food establishments and overall diet quality (i.e., DASH accordance). Given the role that fast-food usage might play in overall diet quality, understanding what personal and structural factors might drive people to different types of food establishments could provide much needed insight into the complex relationships between environmental determinants of diet and health.^44^

Although there is some suggestion that away-from-home eating can be balanced, healthy, and inexpensive,^45^, ^46^ this study demonstrated that at the population level, eating away from home was associated with a poorer diet quality and obesity, likely driven by the use of fast-food outlets. Fast-food outlets often serve large portion sizes for low prices, with meals that tend to be energy dense and nutrient-poor.^47^, ^48^, ^49^ However, the results presented here suggest that users of fast-food outlets may also have overall dietary patterns that are less healthy, regardless of the healthfulness of the meals consumed at any particular food establishment. In fact, consumers of fast food report preferences for food sources that are convenient, easy to access, and provide palatable foods, with availability of nutritious foods often ranked as the least important consideration.^50^ In addition to taste and convenience, fast foods tend to provide a greater calorie-to-cost ratio, which may be particularly important for lower-income shoppers.^51^ It is challenging to separate the potential structural factors that contribute to these preferences: for example the usage of specific food establishments in this study was differentially patterned by SES, with sit-down restaurant and café users being more affluent people.^52^ It has been suggested that individual preferences are socioeconomically patterned, with socioeconomically advantaged individuals possessing more material, psychosocial, and time-related resources, allowing them more easily to make healthier choices related to food retail settings and food items.^53^, ^54^ Therefore, a better understanding of how individual needs and preferences, particularly for different socioeconomic groups, is likely an important area of consideration for any future research or intervention efforts to reduce usage of these food establishments.

### Limitations

This work was a cross-sectional analysis, and therefore these associations may not indicate causality. Additionally the pre-existing categorization of food locations within the NDNS data were primarily based on criteria set out by the NDNS study team, and if modified may yield different results. For example, the fast-food outlets and sit-down restaurants were distinguished by NDNS based on the method of service and did not account for the healthfulness of foods available within food establishments, which may vary substantially^47^ with restaurants providing options that can be as unhealthy as those sold in fast-food outlets.^48^

In addition to characterizing the exposure, a limitation of dietary assessment is that energy intake is often underreported, which can lead to overestimation of the association between exposure and outcome. The use of data from a 4-day food diary reduces underreporting, but it is unlikely to have been eliminated.^55^ In addition, it is not known if underreporting may have been systematically different for different food establishments included in this analysis. Also, the obesity models did not adjust for energy expenditure or level of physical activity, which may be important confounders.

This work also has several strengths, including a prospective measure of away-from-home eating, and restaurant, fast-food, and café usage, overcoming limitations of retrospective measures that are subject to recall bias. Additionally, diet and adiposity outcomes were based on robust methods (4-day diet diaries and objective measurement, respectively) in a nationally representative sample of UK adults. Finally, diet quality was indicated by accordance with the DASH diet, which has strong associations with chronic disease risk.^56^ However, other diet quality indices could also be used to strengthen the interpretation of results presented here. For example, the Mediterranean diet pattern shares some characteristics with DASH, but may result in different patterns of associations with outcomes.^57^

## Conclusions

This study’s findings indicate that while greater reliance on eating away from home is associated with less-healthy diets and obesity, dietary public health interventions designed to modify the usage of food establishments may be most impactful if focused on modifying the usage of fast-food outlets. Further research is required to better understand how and why individuals use specific food establishments, particularly given the complex social, economic, and environmental contexts in which food choices are made.
