# Supplementary material for: Utilization of Away-From-Home Food Establishments, Dietary Approaches to Stop Hypertension Dietary Pattern, and Obesity
Source: Am J Prev Med. 2017 Nov;53(5):e155–63. doi: 10.1016/j.amepre.2017.06.003 (PMC5640390; doi:10.1016/j.amepre.2017.06.003)
Supplement: Supplementary file 1 — Supplementary material [file mmc1.pdf]

SUPPLEMENTARY METHODS

Calculating Eating Occasions and DASH Food Group Scoring and Modifications

In contrast to eating occasions derived strictly on change in time between food items consumed (e.g., ≥15 minutes, or ≥1 hour), derivation of an eating occasion for this analysis was based on larger timeslots, while incorporating change in location within time slots to allow flexibility and reduce the likelihood that eating occasions would be overestimated for away-from-home locations. In particular the locations that are the focus of this work (i.e., where participants are not in control over the timeliness of food service [e.g., sit-down restaurants]).

Initially, each row of data represented a single food item or ingredient consumed by the participant, the total number of food entries was 220,905 over the 4-day recording period. The new variable "eating occasion" (EO) was used to aggregate food items within a single sitting as defined and operationalized above. The total number of EOs across all away-from-home locations generated after aggregation was 12,256 (25.2% of total EOs) of which 12% of EOs occurred in sit-down restaurants, 1% in fast-food outlets, 5% in cafés, and the remaining 82% in non-retail away-from-home locations (e.g., a friend's house) described below.

Eating Location Classifications

Participants recorded the location where each food was consumed as free text in the diet diary (e.g., "McDonalds", "on the train", "at a friend's house", "Costa", etc.). Locations were initially coded and collapsed into 36 subcategories by NDNS. We further collapsed these into five retail-settings: "Restaurants, pubs, and night clubs", "Fast-food and takeaway", "Cafes and sandwich shops", "Other non-retail locations" (all non-home locations were considered "Away-from-home"), and "Home" for this analysis (Appendix Table 1). Although participants provided

details on “Where” eating occasions took place, these categories do not necessarily reflect where food was sourced. Therefore, reallocation of categories was used to better estimate food source using reported eating location. This included reallocating “Work – food from home” from the “Away-from-home” to the “Home” category, and reallocating eating occasions where all food items were appended with a code reflecting “take away” from any category to the “Fast-food and takeaway” category (e.g., takeaway food consumed “on the train” was categorized as “Fast-food and takeaway”). The purpose was to improve the sensitivity of the categories (i.e., avoid false negatives – which is the likely instances of takeaway foods consumed outside of fast-food and takeaway locations). The original coding rules provided by the NDNS team that were applied by trained coders included the following for the food service establishments in this publication:

- Fast-food outlets are distinguished from restaurants by the use of cutlery (e.g., Pizza Hut is a restaurant as they provide cutlery whereas KFC is a fast-food outlet as they do not).
- The distinction between the Restaurant, pub, nightclub option and the Coffee shop, shop, deli, sandwich bar option is that in the former alcohol would be available.

Modifying the DASH Score for NDNS

The score used here is developed from one first used by Fung and colleagues,[58] measuring accordance to the DASH diet based upon the following food groups: fruits, vegetables, nuts and legumes, whole grains, low-fat dairy, sodium, red and processed meats, and sweetened beverages (Appendix Table 2). This score was modified to include all non-milk extrinsic sugars in the diet, whereas in the study by Fung and colleagues this was limited to sugar-sweetened beverages.

Consumption of each food group was adjusted for dietary energy using the residual method and the resulting residuals were used to rank individuals into quintiles (normally distributed without

need for transformation).[39] For each component of the score, a score of 1–5 could be earned based on the quintile of intake, with a 5 indicating higher intake of food groups to encourage. For foods that are discouraged ("–" in table above) this scoring system was reversed so that a score of 5 was given for the lowest intake and 1 for the greatest intake. The overall score had a range of 8 to 40 and higher scores indicate a diet which has greater accordance to the DASH pattern). The continuous DASH score was then divided into quintiles, with diets in the top quintile coded as 1 (DASH-accordant) and lower quintiles coded as 0 (less DASH-accordant).

Sensitivity Analyses and Results

We also defined exposure as using tertile of eating occasions, rather than tertile of energy consumed within eating locations, to examine possible influence of exposure and outcome being derived from diary data.[43] Alternative multivariable model structures for DASH accordance and obesity were explored for robustness. Additional covariate specifications were also examined for robustness, including occupational class (Routine and manual, Intermediate, Higher managerial, administrative and professional, never worked), ethnicity (white, all others), and country of residence (England, Scotland, Ireland, Wales). Additionally, although not the focus of this analysis but used for model adjustment, descriptive tables with ORs and 95% CIs for away-from-home non-retail locations (e.g., friend's house), DASH accordance and obesity were examined.

In sensitivity analyses, no significant differences between the resulting ORs using the two potential exposures, % eating occasions versus % energy intake, across eating locations were found, so energy intake exposure alone was reported. Alternative multivariable models with using occupational class, ethnicity, and country of residence did not significantly alter results (results not shown). Additionally, although not the focus of this analysis, descriptive tables with

ORs and 95% CIs for away-from-home non-retail locations (e.g., friend’s house), DASH accordance and obesity are available in Appendix Table 4, and showed a significant lower odds of DASH accordance for the highest tertile of away-from-home non-retail exposure (OR, 0.68; 95% CI, 0.29–0.58) and no association with odds of obesity.

Appendix Figure 1. Flow diagram to illustrate the sampling strategy used in NDNS.

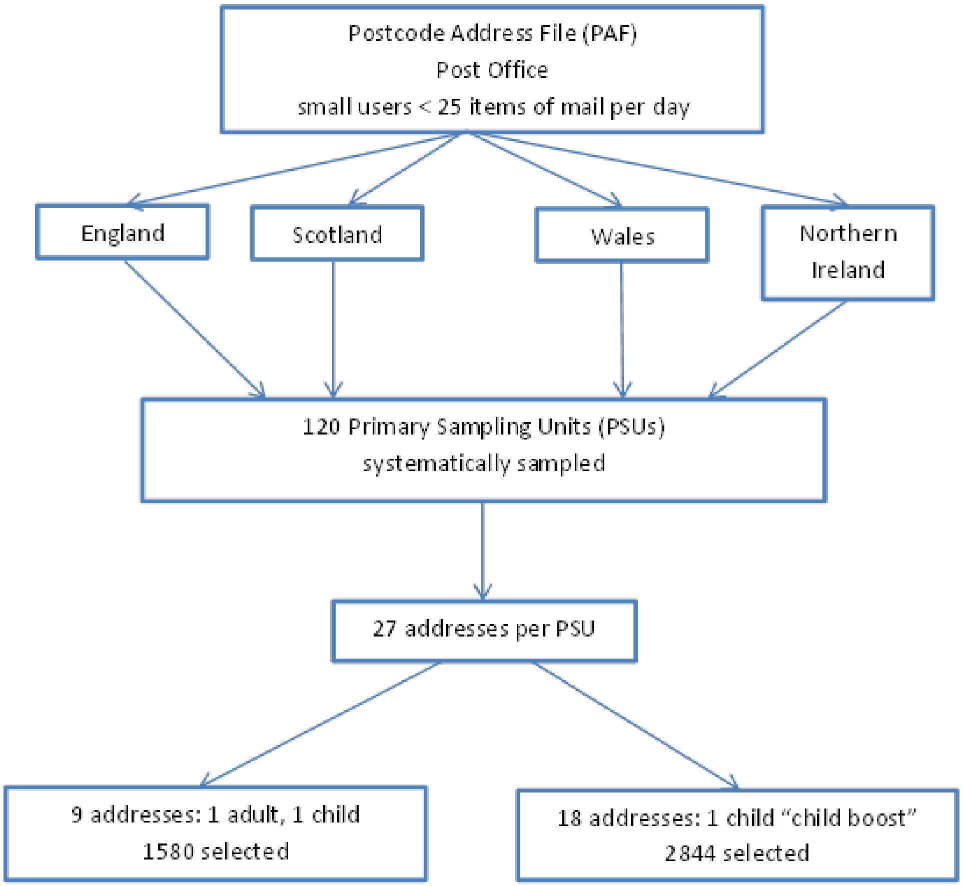

Appendix Table 1. Mapping of NDNS' Original 36 "Where" Categories to Five Eating Location Categories Used in This Study

Study labels/ Aggregated to 5 categories	Original 36 NDNS "Where" categories
Away-from-home food establishments	
Fast-food	Fast-food outlet (e.g., Burger King, Dominos, McDonalds, KFC, burger vans, fish and chip shops)
Sit-down restaurant	Restaurant, pub, night club (e.g., Carluccio's, ASK, Pizza Express, Zizzi, Nando's, Prezzo, Frankie and Benny's, Bella Italia, Café Rouge, TGI Fridays, Hungry Horse, Wetherspoon, Harvester, Toby Carvery, Beefeater, Brewer's Fayre)
Cafés	Coffee shop, cafe, shop, deli, sandwich bar (e.g., Pret a Manger, Greggs, Starbucks, Subway, Costa)
Away-from-home other	
Other	Work - Desk
	Work - Other
	Work - Canteen - Other
	Work - Canteen - Bought food
	School - Classroom
	School - Canteen - Other
	School - Canteen - Bought food
	School - Playground
	School - Other
	Bus, car, train
	Street
	Outside - Other
	Friend's or Relative's house
	Community Centre/Day Centre/Drop-in
	Place of Worship
	Public Hall/Function Room
	Sports club, sports leisure venue
	Leisure Activities, shopping, tourist attractions, cinema, places of interest
	Holiday Accommodation
	Nursery/Kindergarten
	Carer's home
	Not At Home - Unspecified
	Unspecified
	Other place
Within the home	
Home	Home - Living Room
	Home - Kitchen
	Home - Other
	Home - Garden
	Home - Dining Room
	Home - Bedroom
	Home - Unspecified
	Work - Canteen - Food from home
	School - Canteen - Food from home

Appendix Table 2. DASH Food Group Scoring. DASH Accordance Scoring.

**Food group**	**Foods included**	**Scoring**[a]
Fruits[b]	All fresh, dried and tinned fruit, fruit juice, and smoothies	+
Vegetables[b]	All vegetables excluding beans and potatoes	+
Nuts and legumes	All beans,[b] nuts (including peanut butter) and seed	+
Whole grains	Wholegrain bread, wholegrain breakfast cereals, wholegrain pasta, brown rice	+
Low fat dairy products	Low fat yogurt (less than 3% fat), Low fat cheese (less than 3% fat), skimmed milk, 1% fat milk	+
Red and processed meats	All red meat[b] and processed poultry[b]	–
Non-milk extrinsic sugars[b]	All non-milk extrinsic sugars	–
Sodium[b]	Dietary sodium	–

[a]Scoring of "+" indicates food groups that are positively scored; "–" indicates food groups that are negatively scored (i.e., greater consumption of these groups is associated with a lower score).
[b]Indicates that the NDNS reports the amount consumed including when the food appears in composite dishes

Appendix Table 3. Weighted Sample Characteristics for Full Analytic Sample (n=2,083), Percentage of Participants Across Demographic, Socioeconomic, Exposure, and Outcome Measures With Mean (95% CI) Where Indicated

Characteristics	Men	Women	Total
N (unweighted %)	901 (43.2)	1,182 (56.7)	2,083
Demographic [a]			
Age, years	47.1 (45.8–48.4)	48.9 (47.5–50.2)	48.1 (47.1–49.0)
Socioeconomic			
Education			
None, GCSE or equivalent	39.8	44.6	42.3
Further	26.1	25.0	25.5
Degree or higher	25.4	21.8	23.5
Missing	8.6	8.4	8.5
Equivalized household income			
<£14,999	15.2	21.7	18.5
£15,000–£24,999	21.3	20.3	20.8
£25,000–£34,999	17.3	17.0	17.1
£35,000–£49,999	14.6	12.0	13.2
≥£50,000	14.7	11.7	13.2
Missing	16.6	17.1	16.9
% Food outlet usage [a]			
% Restaurant outlet usage	5.38 (4.71–6.06)	5.25 (4.58–5.93)	5.32 (4.81–5.82)
% Fast food outlet usage	4.43 (3.71–5.14)	3.65 (3.14–4.16)	4.03 (3.61–4.45)
% Café food outlet usage	1.19 (0.96–1.41)	1.53 (1.28–1.77)	1.36 (1.21–1.52)
% Other away from home locations	16.8 (15.6–18.1)	15.1 (13.9–16.1)	15.9 (15.1–16.7)
% Total away from home food outlet usage	27.8 (26.2–29.4)	25.5 (24.0–27.0)	26.6 (25.5–27.7)
Diet			
Energy intake (kJ/day)	8,827 (8,622–9,028)	6,646 (6,521–6,767)	7,706 (7,575–7,831)
DASH accordant	13.0	19.9	16.5
Adiposity			
Obesity (BMI ≥30 kg/m^2)	26.2	29.9	28.1

Note: Energy Intake (kcal/day) - Men: 2,109 (2,060–2,157); Women: 1,588 (1,558–1,617); Total: 1,841 (1,810–1,871)
[a]Weighted mean (95% CI)
DASH, Dietary Approaches to Stop Hypertension; Accordant, Highest quintile; GCSE, General Certificate of Secondary Education.

Appendix Table 4. Weighted Sample Characteristics as Percentages (Unless Otherwise Stated) With ORs and 95% CIs for DASH Accordance (n=2,083) and Obesity (n=1,902) by Tertile of Other Away-From-Home Location Usage

**Characteristics**	**Tertile of other away-from-home location usage**		
	**Lowest**	**Middle**	**Highest**
n	692	711	680
Proportion of energy (kJ) (min – max)	0.00 – 0.03	0.03 – 0.20	0.20 – 1.0
Demographics			
Age, years [a]	56.3 (54.7, 57.9)	47.8 (46.3, 49.2)	40.0 (38.7, 41.2)
Sex (% male)	48.3	43.4	53.8
Ethnicity (% white)	88.8	91.3	88.6
Socioeconomics			
Educational attainment (% degree)	16.1	25.4	29.2
Equalized income (% >£35,000)	19.7	33.0	42.1
Occupation (% professional)	35.0	42.95	49.4
Behavior			
Smoking (% never smoked)	50.9	58.6	56.8
Diet			
Fruit and vegetable (g/day) [a]	289 (274,304)	301 (287,314)	277 (262,292)
DASH score (% most accordant)	17.4	19.9	12.4
Energy intake (kJ/day) [a]	7,304 (7,112, 7,501)	7,652 (7,459, 7,844)	8,158 (7,911, 8,405)
Adiposity			
Self-reported health (% very good)	26.2	33.9	41.6
BMI (% normal)	26.0	30.9	35.7
Odds of DASH accordance[b]			
Model 1	1.00 (–)	1.18 (0.85, 1.64)	**0.67** (0.47, 0.95)
Model 2[d]	1.00 (–)	1.35 (0.97, 1.87)	0.85 (0.60, 1.20)
Model 3[e]	1.00 (–)	1.19 (0.84, 1.70)	**0.68** (0.47, 0.98)
Odds of obesity[c]			
Model 1	1.00 (–)	0.84 (0.63, 1.11)	0.82 (0.62, 1.07)
Model 2[d]	1.00 (–)	0.95 (0.71, 1.26)	1.07 (0.80, 1.42)
Model 3[e]	1.00 (–)	1.01 (0.75, 1.35)	1.19 (0.88, 1.59)

Note: Boldface indicates statistical significance (p<0.05).
[a]Weighted Mean % (95% CI)
[b]DASH accordance score was divided into quintiles, with the highest quintile being most DASH accordant.
[c]Obesity included participants with a BMI ≥30 kg/m^2.
[d]Adjusted for age, sex, total energy (kJ), survey year (and smoking status for obesity models).
[e]Additionally adjusted for equivalized income; proportion energy (kJ) from restaurant, fast-food, and café locations.
DASH, Dietary Approaches to Stop Hypertension
